# Widespread Coronary Dysfunction in the Absence of HDL Receptor SR-B1 in an Ischemic Cardiomyopathy Mouse Model

**DOI:** 10.1038/s41598-017-18485-6

**Published:** 2017-12-22

**Authors:** James T. Pearson, Misa Yoshimoto, Yi Ching Chen, Rohullah Sultani, Amanda J. Edgley, Hajime Nakaoka, Makoto Nishida, Keiji Umetani, Mark T. Waddingham, Hui-Ling Jin, Yuan Zhang, Darren J. Kelly, Daryl O. Schwenke, Tadakatsu Inagaki, Hirotsugu Tsuchimochi, Issei Komuro, Shizuya Yamashita, Mikiyasu Shirai

**Affiliations:** 1 Monash Biomedical Imaging Facility, Melbourne, Victoria, Australia; 2 Department of Physiology, Monash University, Melbourne, Victoria, Australia; 3 Australian Synchrotron, Melbourne, Victoria, Australia; 4 National Cerebral and Cardiovascular Center Research Institute, Suita, Osaka, Japan; 5 St Vincent’s Hospital, University of Melbourne, Melbourne, Victoria, Australia; 6 Department of Cardiovascular Medicine, Graduate School of Medicine, Osaka University, Suita, Osaka, Japan; 7 Japan Synchrotron Radiation Research Institute, Harima, Hyogo, Japan; 8 Department of Physiology – HeartOtago, University of Otago, Dunedin, New Zealand; 9 Department of Cardiovascular Medicine, Graduate School of Medicine, University of Tokyo, Tokyo, Japan; 10 Departments of Community Medicine and Cardiovascular Medicine, Graduate School of Medicine, Osaka University, Suita, Osaka, Japan; 11 Rinku General Medical Center, Izumisano, Osaka, Japan; 12 Department of Health Sciences, Nara Women’s University, Nara, Japan

## Abstract

Reduced clearance of lipoproteins by HDL scavenger receptor class B1 (SR-B1) plays an important role in occlusive coronary artery disease. However, it is not clear how much microvascular dysfunction contributes to ischemic cardiomyopathy. Our aim was to determine the distribution of vascular dysfunction *in vivo* in the coronary circulation of male mice after brief exposure to Paigen high fat diet, and whether this vasomotor dysfunction involved nitric oxide (NO) and or endothelium derived hyperpolarization factors (EDHF). We utilised mice with hypomorphic ApoE lipoprotein that lacked SR-B1 (SR-B1^−/−^/ApoER61^h/h^, n = 8) or were heterozygous for SR-B1 (SR-B1^+/−^/ApoER61^h/h^, n = 8) to investigate coronary dilator function with synchrotron microangiography. Partially occlusive stenoses were observed *in vivo* in SR-B1 deficient mice only. Increases in artery-arteriole calibre to acetylcholine and sodium nitroprusside stimulation were absent in SR-B1 deficient mice. Residual dilation to acetylcholine following L-NAME (50 mg/kg) and sodium meclofenamate (3 mg/kg) blockade was present in both mouse groups, except at occlusions, indicating that EDHF was not impaired. We show that SR-B1 deficiency caused impairment of NO-mediated dilation of conductance and microvessels. Our findings also suggest EDHF and prostanoids are important for global perfusion, but ultimately the loss of NO-mediated vasodilation contributes to atherothrombotic progression in ischemic cardiomyopathy.

## Introduction

Occlusive coronary artery disease (CAD) is a leading cause of morbidity and mortality. Heart failure that results from ischemic cardiomyopathy, due to the progression of CAD and or subsequent myocardial infarction, is attributed to remodeling and a weakening of the heart muscle and remains difficult to treat. While it is clear that thrombosis and progressive occlusion by plaque formation of the epicardial large arteries are major causes of myocardial infarction and acute heart failure, it is not clear the extent to which microvascular dysfunction contributes to the progression of ischemic cardiomyopathy.

It is widely recognized that high density lipoprotein (HDL) play an important role in inhibiting the initiation and progression of atherosclerosis through reverse cholesterol transport from cells. HDL also appear to protect the endothelium by promoting activity of HDL-associated antioxidants that inhibit oxidation of low density lipoproteins (LDL)^1–3^ and thereby reduce foam cell initiation. However, high levels of HDL have also been shown to be detrimental, suggesting that HDL alone does not prevent atherosclerosis development, rather that other factors must be required for cardioprotection^3^. The scavenger receptor class B, type I (SR-B1) is the most important regulator of HDL, responsible for selective uptake of cholesterol esters without sequential internalization and degradation of the HDL particles, and results in cholesterol efflux^4^. Deficiency in SR-B1 causes accumulation of large cholesterol ester rich HDL particles, reduced antioxidant activity and elevated *in vivo* isoprostane F2α in plasma, liver and urine as result of oxidation of LDL^3^. Furthermore, the same study showed that systemic oxidative stress was further increased in SR-B1 knockout mice when placed on an atherogenic Paigen diet.

Many transgenic murine models show the progression of atherosclerosis either on a normal chow diet or when challenged on an atherogenic diet. However, most do not develop occlusive CAD. Mice deficient in both SR-B1 and apolipoprotein E (apoE) rapidly develop dyslipidemia, atherosclerosis and spontaneous myocardial infarction on a normal chow diet, resulting in premature death by 6–8 weeks of age^5,6^. Both apoE and SR-B1 are expressed in macrophages which are critical to atherosclerotic lesion formation. Yancey and colleagues^7^ have shown that the greatly accelerated progression of atherosclerosis in these double knockout mice is due to both abnormal plasma lipid levels and severely altered macrophage cholesterol trafficking. In contrast, hypomorphic E mice deficient in SR-B1 with low expression of an allelic variant of mouse apoE (SR-B1^−/−^/ApoeR61^h/h^), have a normal life span on a normal chow diet, but die prematurely from repeated spontaneous myocardial infarction as a result of atherothrombosis after 4–8 weeks on a Paigen diet^8^. Recently, some of us showed that by reducing Paigen diet exposure to 1 week extended survival of SR-B1^−/−^/ApoER61^h/h^ mice, and induced a state of progressive ischemic cardiomyopathy^9^.

Elevated LDL and depressed levels of HDL not only contribute to atherosclerotic plaque formation in large coronary arteries, but also impair endothelial function of all coronary arterial vessels, including the microvessels^10–12^, which do not form atherosclerotic plaques. HDL particle binding to SR-B1 stimulates endothelial nitric oxide synthase (eNOS) activity, nitric oxide (NO) production and endothelium-dependent vasodilation^13–15^. Available evidence suggests that in many vascular beds the endothelial dysfunction associated with hypercholesterolemia and hyperlipidemia involves impairment of NO-mediated dilation, but dilatory prostanoids or endothelium-derived hyperpolarization factors (EDHF) might be more resistant^16–22^. In human biopsies, eNOS protein and mRNA expression tend to be reduced in the endothelium of early lesions (fatty streaks) and significantly reduced in advanced atherosclerotic plaques^23^. In normal coronary arterioles acute infusion with oxidized LDL (oxLDL) greatly reduced endothelium-derived, NO-mediated dilation in a manner similar to that observed in larger atherosclerotic vessels^10,11^. Whether an impairment of vasodilatory capacity of the microvessels drives the pathological progression of ischemic cardiomyopathy associated with CAD due to reduced vasodilator production by the endothelium has not been established.

Current clinical imaging devices are not suitable for *in vivo* assessment of endothelial function in arterial resistance vessel sizes smaller than 100 μm, due to lengthy acquisition times and limited spatial resolution. Using synchrotron radiation microangiography we have recently developed novel methods to repeatedly visualize the resistance vessels of the microcirculation (30–100 μm, small arteries-arterioles) *in vivo* and quantify regional differences in calibre under conditions of high heart rate (>500 bpm)^24–27^. In this study, our aim was to investigate the early origins of vascular dysfunction across individual coronary microvessel segments *in situ* in anaesthetized SR-B1^−/−^/ApoER61^h/h^ mice previously exposed to a short-period of the atherogenic Paigen diet. We tested the hypothesis that the development of atherosclerosis and ischemic cardiomyopathy in the coronary macro and microcirculations involves impaired NO-mediated dilation, but not EDHF.

## Results

### Animal Characteristics, Structural Changes and Oxidative Stress

**Figure 1:**
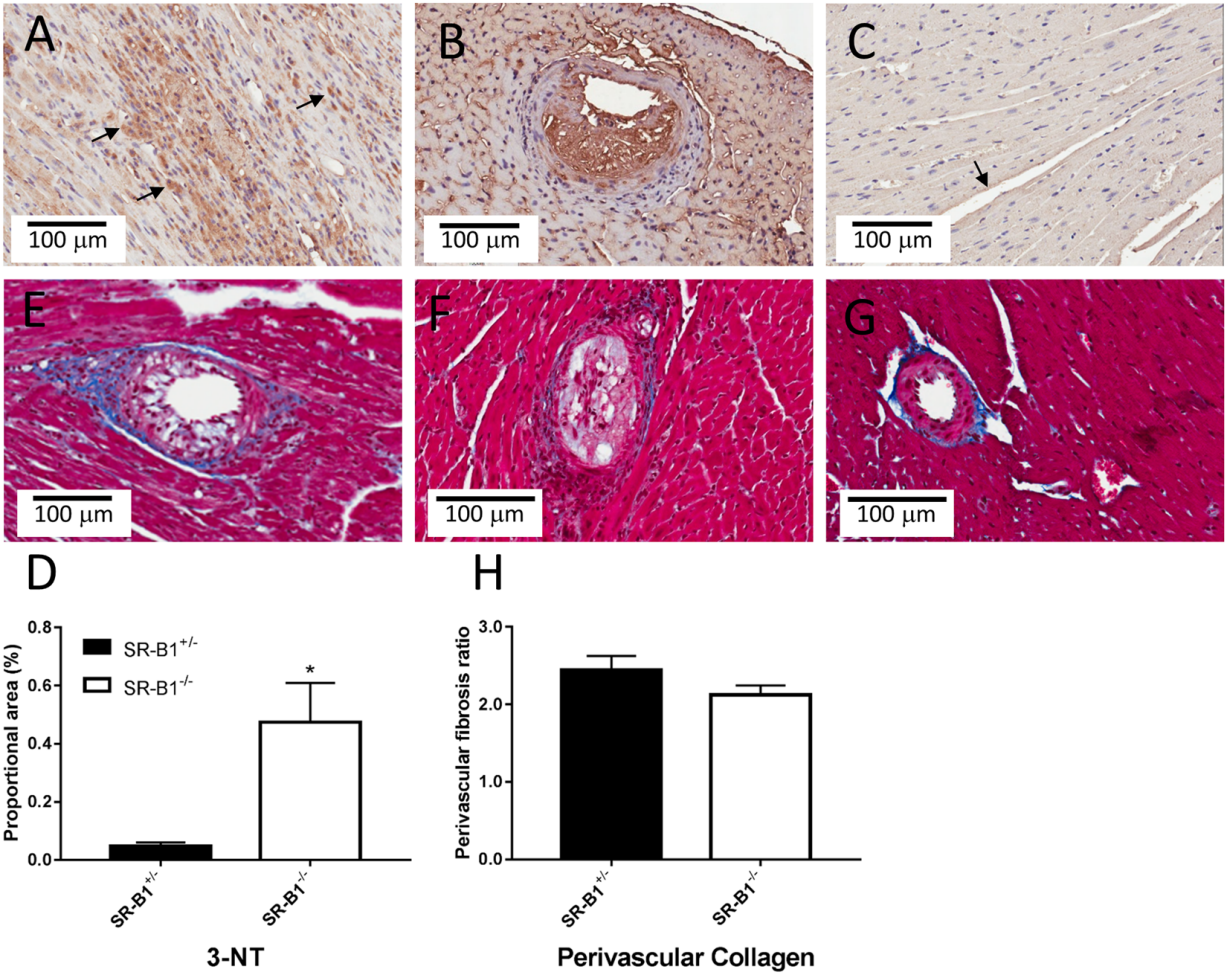
Myocardial nitrotyrosine and perivascular collagen expression after brief Paigen diet exposure. Representative immunohistochemical staining for nitrotyrosine expression in SR-B1^−/−^/ApoER61^h/h^ (**A** and **B**) and SR-B1^+/−^/ApoER61^h/h^ (**C**) mice (20X objective). Nitrotyrosine expression was elevated in SR-B1^−/−^/ApoER61^h/h^ mice 5 times that in SR-B1^+/−^/ApoER61^h/h^ mice (**D**, p < 0.05). Perivascular regions stained with Masson’s trichrome in SR-B1^−/−^/ApoER61^h/h^ (**E** and **F**) and SR-B1^+/−^/ApoER61^h/h^ (**G**) mice (20X objective). Collagen expression, evident as blue-stained fibrils, did not differ significantly between mouse groups (**H**). Extensive nitrotyrosine staining was noted in atherosclerotic lesions in SR-B1^−/−^/ApoER61^h/h^ mice only (**B**). SR-B1^−/−^/ApoER61^h/h^, n = 8 and SR-B1^+/−^/ApoER61^h/h^, n = 8. Values are expressed as mean ± SEM.

**Figure 2:**
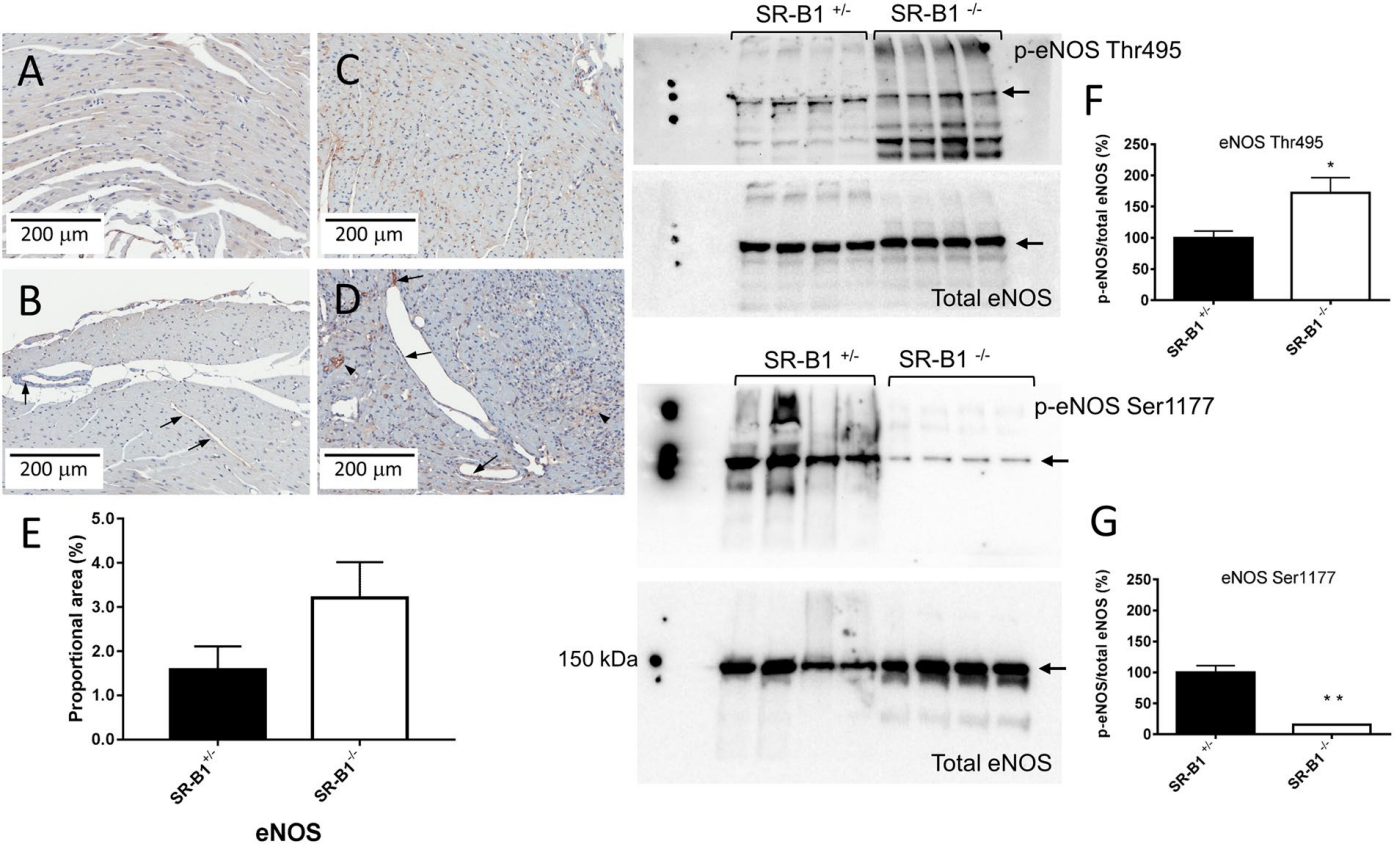
eNOS expression in the myocardium and endothelium. Representative immunohistochemical staining for eNOS expression in SR-B1^+/−^/ApoER61^h/h^ (**A** and **B**) and SR-B1^−/−^/ApoER61^h/h^ (**C** and **D**) mice (20X objective). Epicardial regions highlighting macro and microvessels with endothelium staining for eNOS (arrows) in SR-B1^+/−^/ApoER61^h/h^ and SR-B1^−/−^/ApoER61^h/h^ mice respectively (**B** and **D**). Mean myocardial immunohistochemical eNOS expression did not differ significantly between SR-B1^−/−^/ApoER61^h/h^ (SR-B1^−/−^) and SR-B1^+/−^/ApoER61^h/h^ (SR-B1^−/−^) mice (**E**). Arrow heads denote foam cell lesions with eNOS staining. SR-B1^−/−^/ApoER61^h/h^, n = 8 and SR-B1^+/−^/ApoER61^h/h^, n = 8. Full-length Western blots of myocardial p-eNOS Thr495 and total eNOS and the densitometric analysis of the p-eNOS to total eNOS ratio (**F**), n = 4. Full-length Western blots of myocardial p-eNOS Ser1177 and total eNOS and the densitometric analysis of the p-eNOS to total eNOS ratio (**G**), n = 4. Values are expressed as mean ± SEM. *p < 0.05, **p < 0.01 vs SR-B1^+/−^.

SR-B1^−/−^/ApoER61^h/h^ mice did not differ significantly from SR-B1^+/−^/ApoER61^h/h^ mice in body mass at the time of imaging (29.1 ± 0.6 *vs* 28.9 ± 0.6 g NS), but showed larger relative heart mass (6.60 ± 0.43 *vs* 4.09 ± 0.20 mg/g body weight, p < 0.001). There was no significant difference in perivascular fibrosis in non-occluded vessels (NS, Fig. 1). Nitrotyrosine levels were more than five times higher in SR-B1^−/−^/ApoER61^h/h^ myocardium than in SR-B1^+/−^/ApoER61^h/h^ (p < 0.001) and frequently elevated in both normal myocardial regions and remodeled regions associated with an occluded vessel in the hearts of SR-B1^−/−^/ApoER61^h/h^ mice after exposure to the Paigen diet (Fig. 1). Expression of eNOS protein was clearly evident as a dark brown stain in the endothelium lining of vessels from large coronary arteries to capillaries in both mouse groups (Fig. 2), and there was a non-significant tendency (p = 0.12) for mean myocardial eNOS expression to be higher in SR-B1^−/−^/ApoER61^h/h^ mice. Moreover, myocardial phosphorylation at the Thr495 site of eNOS was significantly upregulated (Fig. 2F) and that of Ser1175 site downregulated (Fig. 2G) in SR-B1^−/−^/ApoER61^h/h^ mice. Therefore, myocardial oxidative stress and eNOS uncoupling, but not perivascular fibrosis likely contributes to coronary dysfunction in SR-B1^−/−^/ApoER61^h/h^ mice.

### Baseline Vessel Number and Internal Diameter

**Figure 3:**
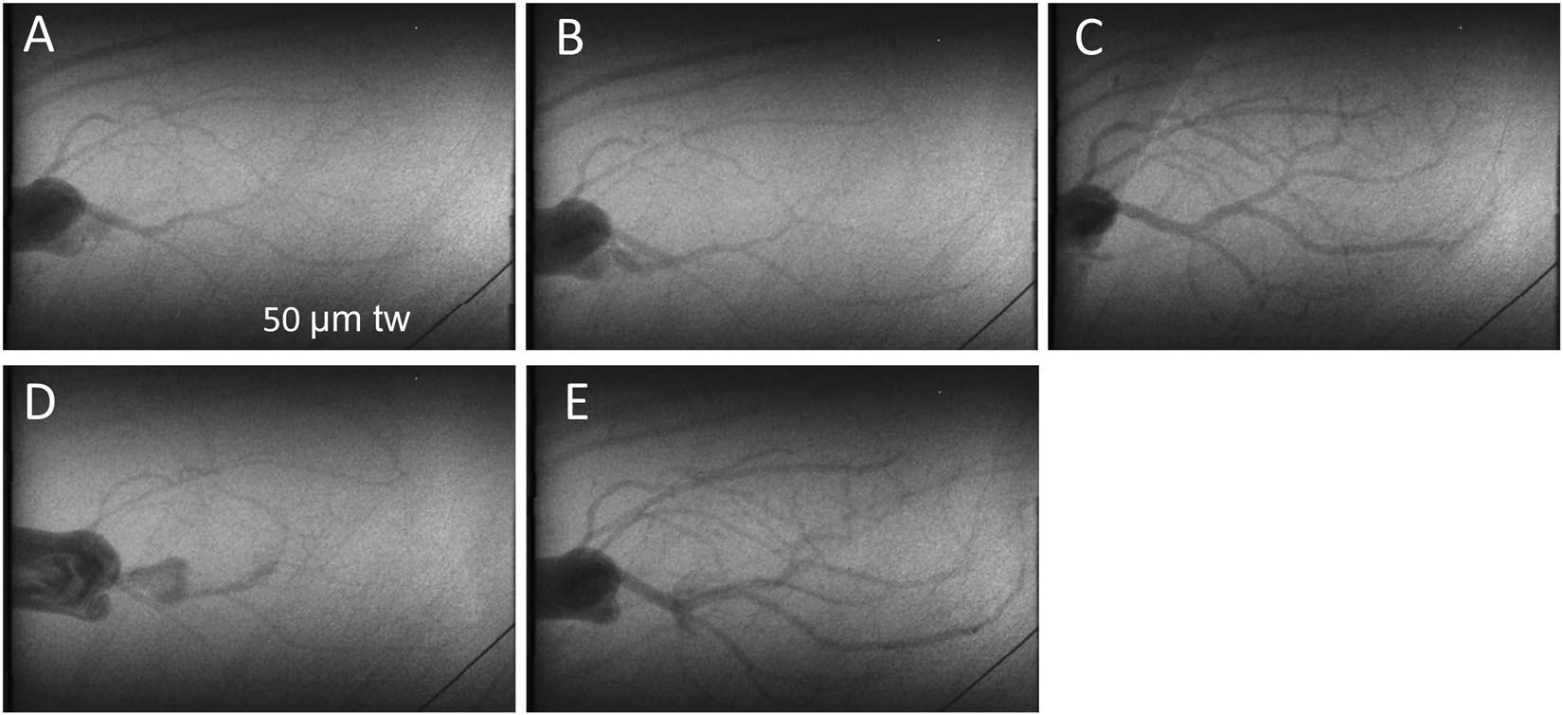
Representative synchrotron radiation angiograms of the coronary vasculature in a SR-B1^+/−^/ApoER61^h/h^ mouse. Baseline response to infusion of vehicle lactate Ringer’s solution (**A**). Distal segments dilated during ACh infusion while large vessels maintained ID similar to baseline (**B**). Large dilation and recruitment of new vessels during SNP infusion (**C**). Widespread constriction following administration of L-NAME and meclofenamate (NOS/COX blockade) (**D**). Pronounced residual dilation in large and small arteries to ACh following NOS/COX blockade (**E**). tw is a 50 μm tungsten wire for vessel ID determination.

**Figure 4:**
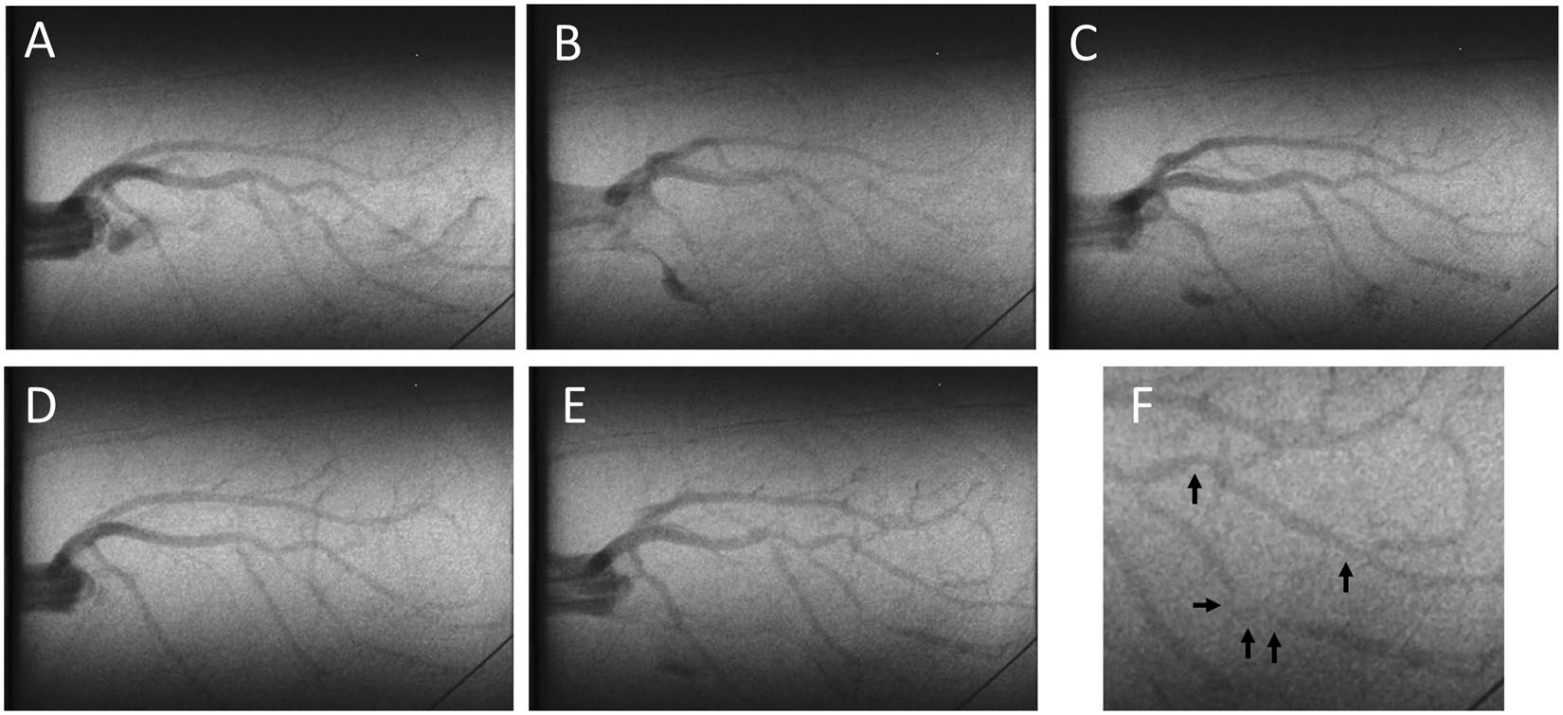
Representative synchrotron radiation angiograms of the coronary vasculature in a SR-B1^−/−^/ApoER61^h/h^ mouse. (**A**) Baseline response to infusion of vehicle lactate Ringer’s solution (**A**). Large vessels maintained ID similar to baseline during ACh infusion, but showed distal ID reduction (**B**). Weak dilation of some distal microvessel segments during SNP infusion relative to baseline (**C**). Modest constriction in some segments following administration of L-NAME and meclofenamate (NOS/COX blockade) (**D**). Dilation in large and small arteries during ACh infusion following NOS/COX blockade, except at localised segments showing constrictions (**E**). Higher magnification of the constricted segments with occlusions (black arrows) shown in E (**F**). Subsequent histology noted atherosclerotic plaques in regions showing constriction (not shown).

**Figure 5:**
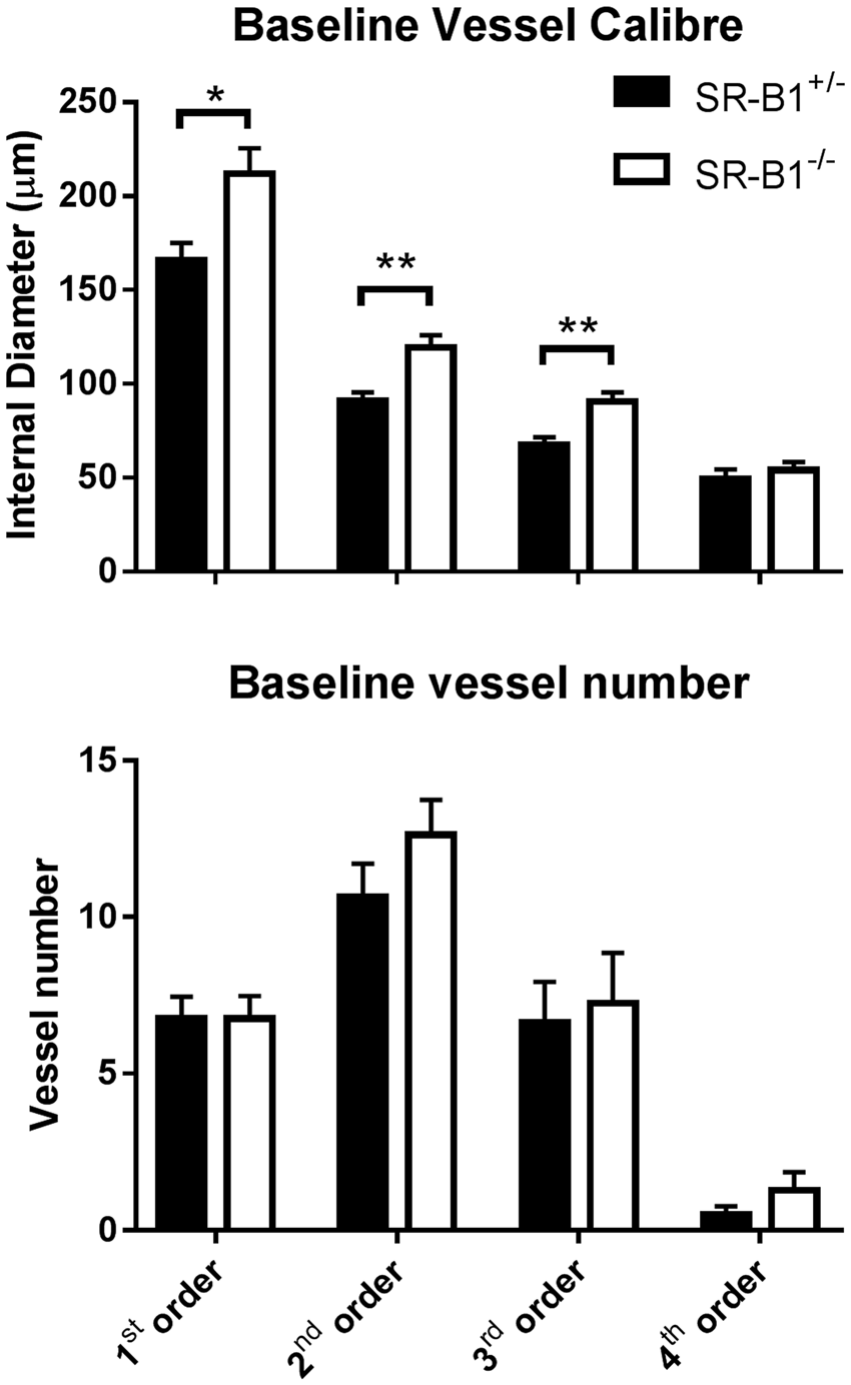
Vessel internal diameter and visible vessel number at baseline in relation to branching order. Vessel ID was significantly larger in SR-B1^−/−^/ApoER61^h/h^ mice in 1^st^, 2^nd^ and 3^rd^ order vessels during baseline, but did not differ significantly in angiographically visible vessel number. SR-B1^−/−^/ApoER61^h/h^, n = 8 and SR-B1^+/−^/ApoER61^h/h^, n = 8. Values expressed as mean ± SEM. *p < 0.05, **p < 0.01 vs. SR-B1^+/−^/ApoER61^h/h^.

**Figure 6:**
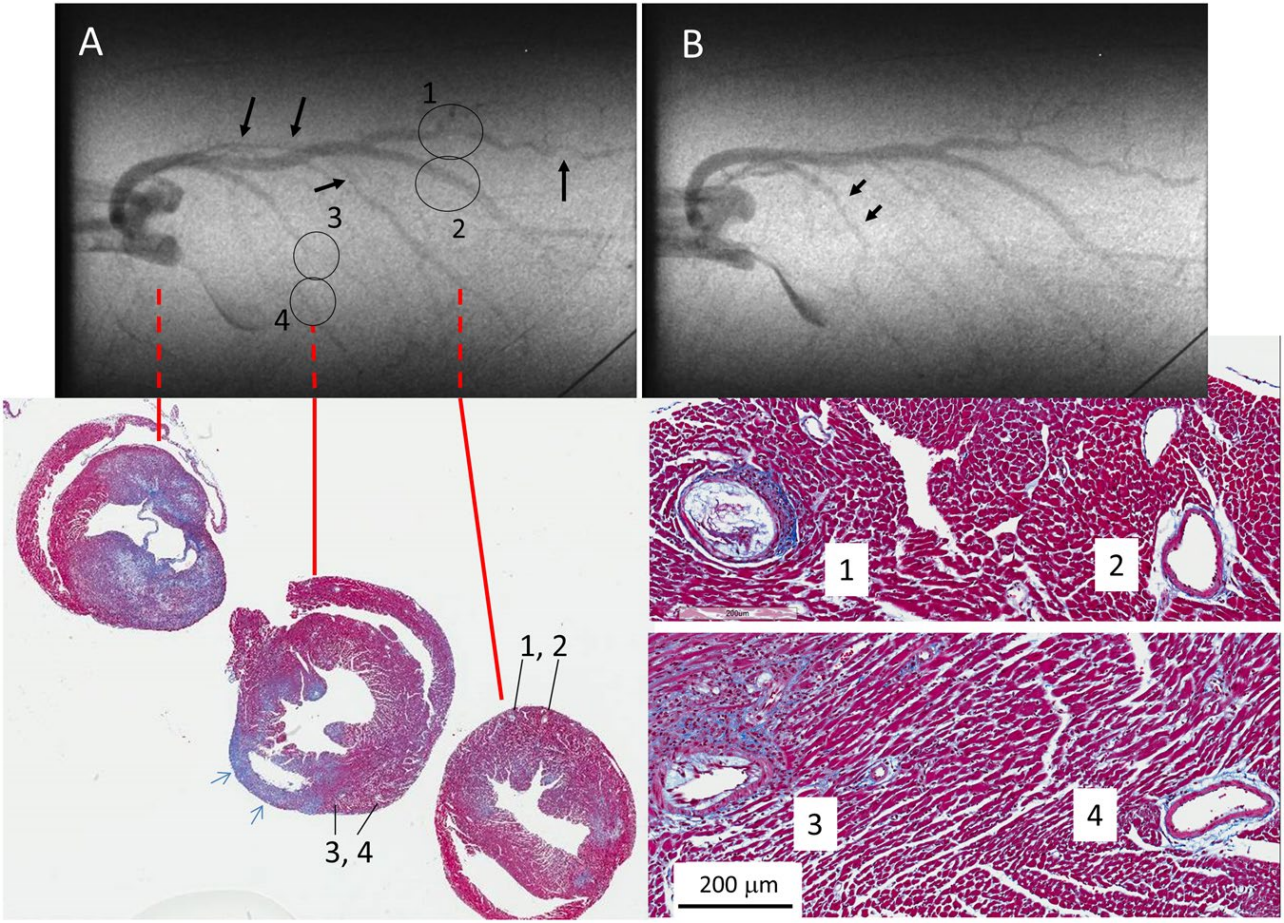
Cine-frames from a SR-B1^−/−^/ApoER61^h/h^ mouse showing multiple stenoses that were confirmed by histological inspection. Consecutive angiogram frames during baseline imaging show extensive stenotic regions. Inset histology panels show the largely occluded lumen (1) and an open artery (2) associated with the left coronary artery and smaller stenosis (3) and open artery (4) associated with the upper left ventricle. Histology panels also show extensive interstitial fibrosis (Masson’s trichrome), and a midwall rupture of the lateral wall (blue arrows). An extensive infarct surrounded the aortic root and base of the left ventricle. Black arrows in A indicate constrictions along the length of a main artery segment that are likely due to partial occlusion by plaques (which also included segment 3).

Representative coronary angiogram frames from cine recordings of SR-B1^+/−^/ApoER61^h/h^ and SR-B1^−/−^/ApoER61^h/h^ mice are presented for baseline and treatment periods (Figs 3 and 4 and Suppl. Fig. S3). A similar number of arterial vessel segments were visualized in the hearts of both groups, across each of the first 4 branching orders of arterial vessels under baseline conditions (Group NS, Fig. 5). Vessel ID in SR-B1^−/−^/ApoER61^h/h^ mice were significantly larger in the first three orders of branching segments (Group and Branching Order p < 0.001), but not the 4^th^ order vessels compared to SR-B1^+/−^/ApoER61^h/h^ mice (Fig. 5). Few 4^th^ order arterioles were visualized in either group during the vehicle infusion. Hence, SR-B1^−/−^/ApoER61^h/h^ mice maintained a greater state of baseline dilation in the large and small conduit coronary arteries, and the larger resistance vessels. In SR-B1^−/−^/ApoER61^h/h^ mice multiple stenoses were visible in large and small artery segments even during baseline imaging (Fig. 6 and Suppl. Fig. S3).

### Baseline Haemodynamics

Mean arterial pressure (MAP) and mean heart rate (MHR), measured at baseline imaging, were not significantly different in SR-B1^−/−^/ApoER61^h/h^ mice from SR-B1^+/−^/ApoER61^h/h^, but MAP was a slightly depressed (59.7 ± 5.0 *vs* 72.8 ± 5.5 mmHg, NS) while heart rate was comparable (417.0 ± 26.4 *vs* 431.0 ± 29.6 bpm, NS) (Suppl. Fig. S4, upper panel).

### Vessel Response to Endothelium- Dependent/Independent Stimulation

**Figure 7:**
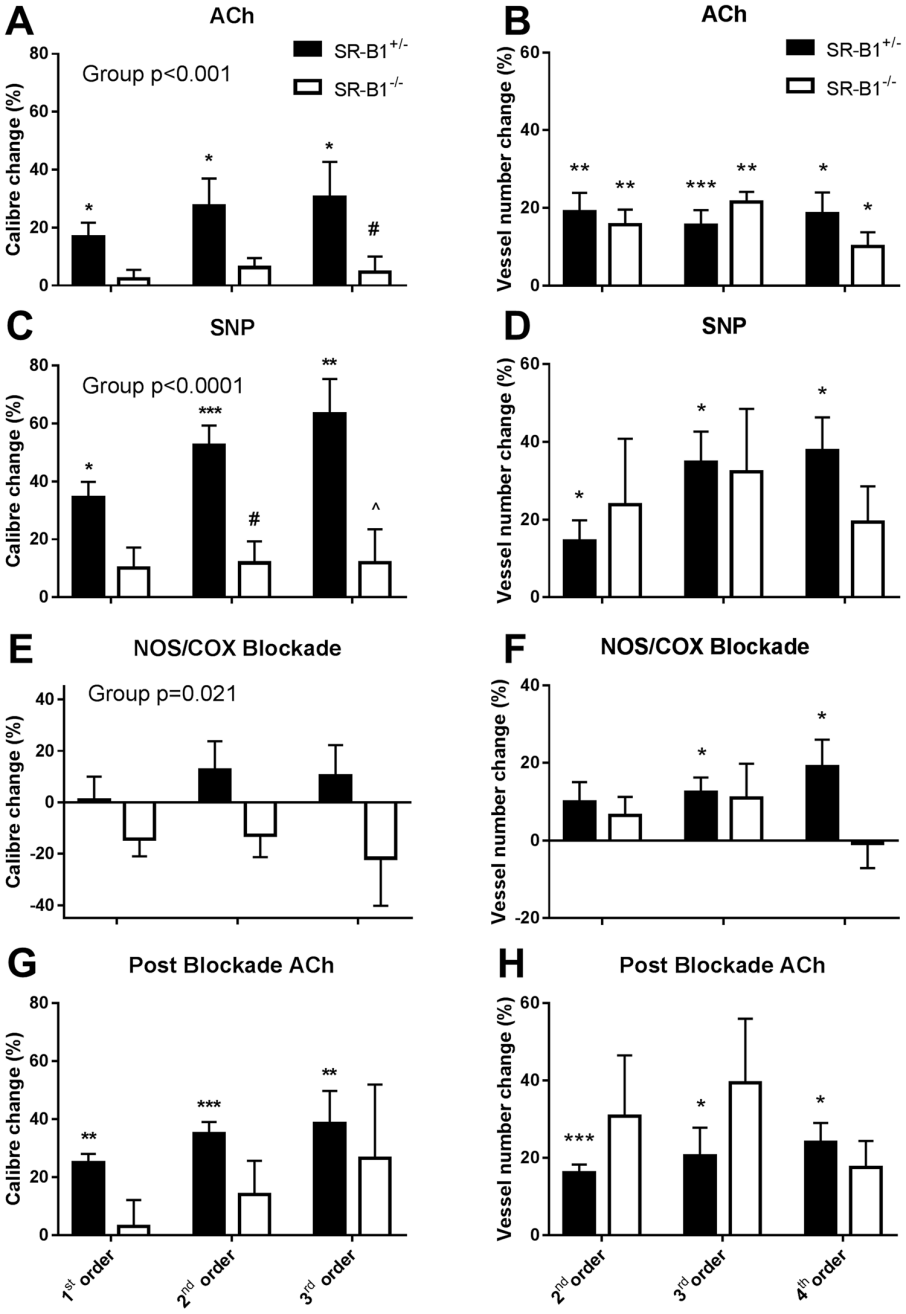
Change in vessel internal diameter and visualized vessel number during infusion of vasoactive compounds. Significant differences in the change in ID from baseline were noted between SR-B1^−/−^/ApoER61^h/h^ and SR-B1^+/−^/ApoER61^h/h^ in response to ACh (**A**) and SNP (**C**) in relation to vessel branching order. However, ACh evoked some residual dilation in SR-B1^−/−^/ApoER61^h/h^ mice following blockade with L-NAME+ meclofenamate relative to the baseline (**G**). No significant differences in visualized vessel number in relation to branching order were noted between SR-B1^−/−^/ApoER61^h/h^ and SR-B1^+/−^/ApoER61^h/h^ responses to ACh (**B**), SNP (**D**) and following blockade with L-NAME+ meclofenamate (**F**) relative to the baseline. No significant differences between groups during ACh post blockade relative to blockade alone (**H**). SR-B1^+/−^/ApoER61^h/h^, n = 8 and SR-B1^−/−^/ApoER61^h/h^ n = 8. Values expressed as mean ± SEM. Data were analysed by 2-way ANOVA. *p < 0.05, **p < 0.01, ***p < 0.001 vs. baseline ID, ^#^p < 0.05, ^p < 0.01 vs. SR-B1^+/−^/ApoER61^h/h^.

Before NOS and COX blockade, endothelium-dependent dilatory responses were only evident in SR-B1^+/−^/ApoER61^h/h^ mice during infusion of ACh, evoking an 18–30% increase in calibre across the first three branching orders (all p < 0.05) (Fig. 7); 4^th^ order of branching segments were omitted due to the low number of vessels for ID determination. Calibre changes during ACh and SNP infusions were significantly different between mouse groups (Fig. 7). The calibre of all arterial vessels in the SR-B1^−/−^/ApoER61^h/h^ mice during ACh infusion did not differ on average from the baseline (Fig. 7A). Nonetheless, MAP decreased by a similar degree in both groups in response to ACh (−22.3 *vs* −29.6 mm Hg, Suppl. Fig. S4). Endothelium-independent dilation in vessels in response to SNP infusion was pronounced in SR-B1^+/−^/ApoER61^h/h^ mice (Fig. 3), with the largest mean increase in 2^nd^ and 3^rd^ order vessels (p < 0.001, Fig. 7C). Similar to ACh, SNP did not evoke a consistent dilatory response in SR-B1^−/−^/ApoER61^h/h^ mice (NS from baseline, Figs 3 and 7C); although regional increases were evident in a few mice (Figs 3 and S5, which contrasted with the vasoconstriction evident in Suppl. Fig.S3). The lack of coronary endothelium-independent responses in SR-B1^−/−^/ApoER61^h/h^ mice was matched by the absence of a decrease in MAP in SR-B1^−/−^/ApoER61^h/h^ mice during SNP infusion, in contrast to SR-B1^+/−^/ApoER61^h/h^ mice (Fig. 7 & Suppl. Fig. S4).

### Vessel Response during NOS/COX Inhibition and subsequent ACh

Blockade of NOS and COX significantly increased MAP in all mice at the end of the 30 min equilibration period, but the increase in SR-B1^+/−^/ApoER61^h/h^ mice was double that in SR-B1^−/−^/ApoER61^h/h^ mice (47.6 ± 6.0 *vs* 20.8 ± 8.3 mm Hg, trend only p = 0.075, Suppl. Fig. S4). However, the decrease in MAP during post blockade ACh infusion resulted in a similar level of MAP during imaging (12–18 mm Hg below the baseline MAP). Blockade resulted in a significant constriction across all branching orders in SR-B1^−/−^/ApoER61^h/h^ mice relative SR-B1^+/−^/ApoER61^h/h^ mice (Fig. 7E). ACh-mediated vasodilation post NOS and COX blockade was slightly potentiated across medium and small vessel branching orders in both mouse groups compared to baseline and the first ACh infusion (Fig. 7G). In SR-B1^−/−^/ApoER61^h/h^ mice, ACh increased microvessel calibre when NOS and COX were inhibited on average, but not consistently so, and the calibre increase in the 2^nd^ and 3^rd^ order branches were not different from SR-B1^+/−^/ApoER61^h/h^ mice.

### Opening of Vessels during Endothelium Stimulation

Despite the absence of significant calibre increases through ACh and SNP-mediated local dilation in SR-B1^−/−^/ApoER61^h/h^ mice there were similar increases in visualized vessel number across 2^nd^, 3^rd^ and 4^th^ branching orders in both groups during ACh (15.6 ± 3.9% to 18.9 ± 3.4% *vs*. 8.8 ± 3.4% to 21.5 ± 2.7%, NS) and SNP infusions (32.5 ± 5.4% to 61.4 ± 11.6%), although SR-B1^−/−^/ApoER61^h/h^ mice were very variable in response to SNP (Fig. 7D). There was no difference between mouse groups in vessel number change from baseline following NOS/COX blockade or relative to blockade during subsequent ACh (Fig. 7F and H).

### Stenoses and Localized Vascular Constrictions in SR-B1^−/−^/ApoER61^h/h^ mice

**Figure 8:**
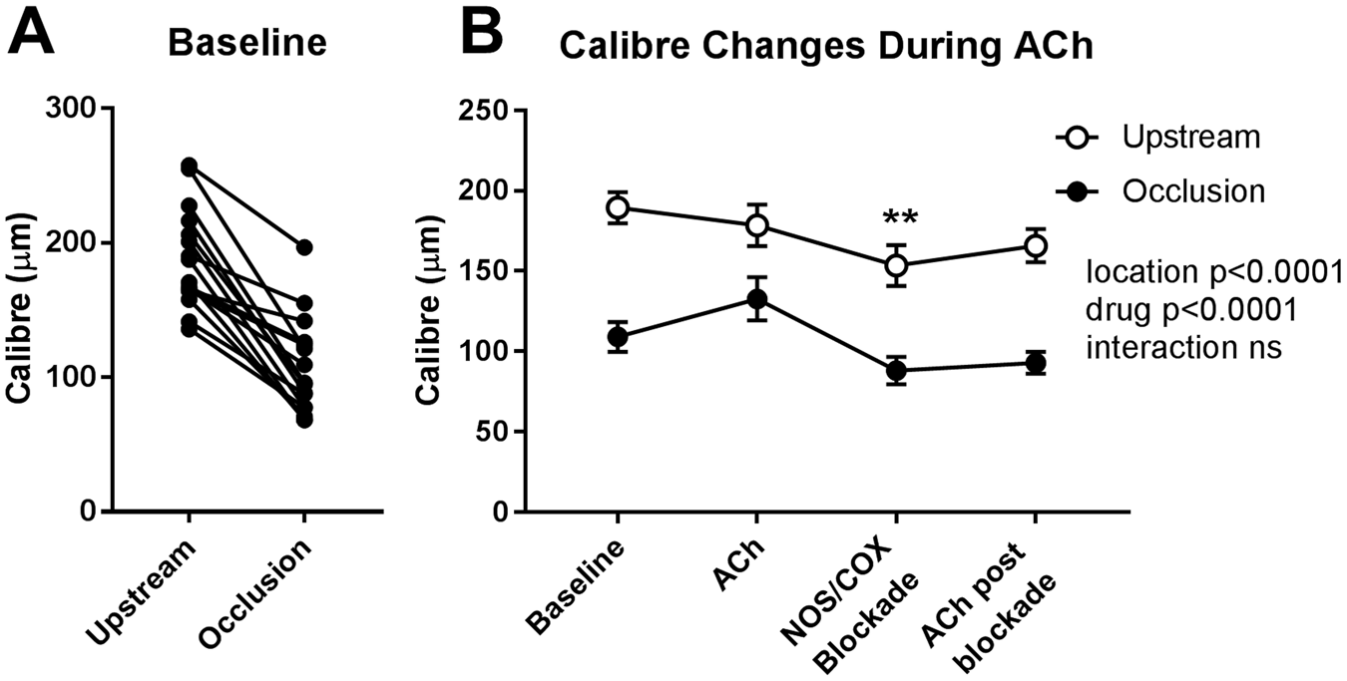
Change in vessel internal diameter at stenosis sites relative to upstream segments at baseline and during ACh stimulation in SR-B1^−/−^/ApoER61^h/h^ mice. Changes in vessel ID at identified stenoses and the adjacent upstream segment region during ACh infusion before and after NOS/COX blockade. n = 4 mice. Occlusion sites and adjacent upstream segments showed significantly smaller calibre after blockade. Dilator responses to ACh were absent in both locations. **p < 0.01 vs. baseline.

No stenoses or regional constrictions were observed in any of the SR-B1^+/−^/ApoER61^h/h^ mice. In contrast, stenoses and constrictions in SR-B1^−/−^/ApoER61^h/h^ mice were most evident during ACh infusion after NOS/COX blockade (Fig. 4E; black arrows), but were also sometimes clearly observed during baseline imaging (Fig. 6 and Suppl. Fig. S3). Notably, severe stenoses were observed in angiograms in 1^st^, 2^nd^ and larger 3^rd^ order segments, often at branching points, but not in smaller segments. Coronary plaque stenoses in SR-B1^−/−^/ApoER61^h/h^ mice were confirmed by histological inspection (Fig. 5 and Suppl. Fig. S3; black arrows). A comparison of vessel ID changes at stenosis sites (including those identified in Figs 4 and 6 and Suppl. Fig. S3) with adjacent upstream sites of the same segments in 4 SR-B1^−/−^/ApoER61^h/h^ mice was made of the ACh responses before and after NOS/COX blockade (Fig. 8). Occlusions in first and second order vessels (>100 μm) were 57.3 ± 4.3% (range 40–86%, n = 16 vessels) of the upstream ID at baseline. Mean calibre of the upstream segments and within the occlusions showed little change in response to ACh before or after blockade, but was significantly more constricted following blockade (Fig. 8). These findings suggest that the upstream segments of stenoses lacked both NO and EDHF-mediated dilator responses when the stenosis appreciably occluded the vessel lumen. Further, in contrast to the open vessel segments of the wider coronary circulation, EDHF was impaired at largely occluded segments in the SR-B1^−/−^/ApoER61^h/h^ mice.

## Discussion

This study investigated whether impairment of the NO and or EDHF-mediated vasodilatory capacity of the coronary microvessels is an important contributor to the pathological progression of ischemic cardiomyopathy. The regulation of coronary perfusion in an ischemic cardiomyopathy mouse model of CAD was examined using microangiography with synchrotron radiation *in vivo* following brief exposure for one week to hypercholesterolemia and dyslipidemia. This within-animal imaging approach revealed several novel findings concerning the coronary dysfunction that evolves following the Paigen diet challenge in the SR-B1 receptor deficient hypomorphic ApoE mouse (SR-B1^−/−^/ApoER61^h/h^). First, widespread vascular dysfunction was evident due to impairment of NO-mediated dilation that was associated with elevated nitrosative stress and eNOS uncoupling. Impairment of local dilation mechanisms, as determined by vessel calibre, was shown to be throughout a large portion of the non-occluded vessels in SR-B1^−/−^/ApoER61^h/h^ mice, including the coronary microcirculation. Second, while local NO-mediated regulation of vessel calibre of the macro and microvessels visualized at baseline was greatly diminished, the ability to increase perfusion through increases in perfused vessel number was maintained in SR-B1^−/−^/ApoER61^h/h^ mice. Since ACh increased visible vessel number in the absence of NO and dilator prostanoids, the residual dilation in the non-occluded vessels is assumed to be EDHF-mediated and therefore, EDHF was largely preserved in these regions of the coronary circulation. Since the impaired response to SNP revealed that NO-mediated signaling is impaired in SR-B1 deficient mice, the widespread constriction following NOS/COX blockade implies that vasodilatory prostanoids must play a compensatory role in the maintenance of coronary perfusion in open segments as well. However, the absence of residual dilation to ACh during blockade, or even constriction, at both the angiographically visible stenotic segments and their adjacent upstream segments in the hearts of SR-B1^−/−^/ApoER61^h/h^ mice revealed localized deficits in EDHF production. Consequently, depletion of NO bioavailability and deficits in production of EDHF likely contribute to localized constriction of occluded segments at baseline. Taking these findings together, deterioration in NO-mediated regulation of vascular function in the main coronary branches and microvessels that maintain basal myocardial perfusion is a key event leading to the development of occlusive lesions and ischemic cardiomyopathy in the absence of SR-B1 receptors. Loss of EDHF appears to be associated with the formation of occlusive plaques.

In this study, SR-B1^+/−^/ApoER61^h/h^ mice with partially reduced SR-B1 receptor expression showed strong local dilator responses in terms of calibre increases to ACh and SNP, revealing that endothelial function was well maintained after exposure to the Paigen diet for one week (Fig. 7). The release of NO from the endothelium involves both PGI_2_-dependent and independent mechanisms in the murine heart^20^. Importantly, in many species endothelium-mediated residual dilation to ACh is observed in the absence of both NO and vasodilator prostanoids. Selective inhibition of diffusible factors and K channels has been used to demonstrate that the residual dilation present during endothelium stimulation following NOS/COX blockade is mediated by multiple putative EDHF^28–32^. NO-mediated local dilation in the SR-B1^+/−^/ApoER61^h/h^ mice was evident in all coronary branching orders, but was most pronounced in small arteries and larger arterioles, while vessel recruitment by NO was primarily observed in small arteries, large and small arterioles (Fig. 7). Residual dilation to ACh during NOS/COX blockade in SR-B1^+/−^/ApoER61^h/h^ mice dilated all vessels significantly compared to baseline except 4^th^ order resistance vessels, indicating that a strong EDHF-mediated dilation capacity is widespread across the vasculature. While the general absence of dilation in smaller arterioles is surprising, there was a 20% increase in the visualized number of arterioles suggesting that coronary flow in the microcirculation was increased *in vivo* in the SR-B1^+/−^/ApoER61^h/h^ mice. Since few of these vessels were visualized at baseline it is likely that a high vascular resistance reduced vessel calibre below the ~30 μm ID resolving ability of our microangiograms^27^. Alternatively, but less likely, contrast agent entering the 4^th^ order vessels was diluted to close to background levels of X-ray absorption. A limitation of this study that must be acknowledged is that we are unable to establish if the level of SR-B1 expression in the endothelial cells of the coronary microvessels influenced these endothelium-mediated dilator responses, due to the small size of the vessels. Nevertheless, this study supports the idea that partial SR-B1 expression is protective of endothelial function following activation of pro-thrombotic and pro-inflammatory pathways after exposure to an atherogenic diet^9,33^, in part due to the preservation of NO-mediated vasodilation.

In this study, we found that SR-B1^−/−^/ApoER61^h/h^ mice bearing multiple diffuse coronary lesions showed evidence of widespread impaired local vascular smooth muscle function characterized by greatly diminished radial transmission of dilation in the non-occluded vessels. This is in stark contrast to SR-B1^+/−^/ApoER61^h/h^ mice that did not form atherosclerotic plaques and maintained potent vasodilatory responses. Various studies suggest that hypercholesterolemia induces endothelial dysfunction, which is characterized by greater impairment of NO-mediated, endothelium-derived relaxation than EDHF-mediated relaxation, and mostly in the large coronary arteries^17,20,22,28^. Impaired NO/cGMP signaling and vascular smooth muscle dysfunction is considered to be a characteristic of the late stage of atherosclerotic lesions^34,35^. However, this and other studies suggest that vascular dysfunction caused by exposure to an atherogenic diet, or even acute exposure to LDLs, is not limited to conduit vessels and can involve smooth muscle dysfunction. Hein and associates^11^ clearly demonstrated in normal coronary arterioles isolated from pigs that even acute exposure to oxidized LDL (oxLDL) impairs endothelium-dependent, NO-mediated dilation by reducing NO biosynthesis and its bioavailability. SR-B1^−/−^/ApoER61^h/h^ mice did not respond to stimulation with ACh or a NO donor with an increase in vessel calibre on average. We reason that this local dilator impairment that we observed in SR-B1^−/−^/ApoER61^h/h^ mice might involve both reduced endothelium release of NO and impaired NO-mediated hyperpolarization of adjacent vascular smooth muscle cells in both conduit and resistance microvessels. Importantly, smooth muscle dysfunction was present in the macro vessels and larger resistance microvessels that are routinely open to maintain basal myocardial perfusion.

It is important to note that impaired regulation of vessel calibre in the vessels involved in maintenance of basal perfusion was not associated with downregulation of eNOS protein expression in SR-B1^−/−^/ApoER61^h/h^ mice subsequent to exposure to the atherogenic diet. Rather, we observed uncoupling of eNOS from NO production due to exposure to the Paigen diet in SR-B1^−/−^/ApoER61^h/h^ mice as the Ser1175 phosphorylation site was hypophosphorylated and Thr495 site hyperphosphorylated relative to that in SR-B1^+/−^/ApoER61^h/h^ mice (Fig. 2). Although eNOS is expressed predominantly in the endothelium, we cannot discount that eNOS uncoupling might also reflect changes in eNOS signaling at the cardiomyocyte plasma membranes. Furthermore, our findings also suggest that reduced effectiveness of the soluble guanylate-cGMP signaling pathway also contributes to this early dysfunction since endothelium-independent stimulation with SNP did not increase vessel calibre. Nonetheless, eNOS expression has been reported to be downregulated during ongoing exposure to an atherogenic diet, in regions of advanced lesions^23,36^. Indeed, in regions of the aorta with disturbed, non-laminar flow that are prone to atherosclerotic lesion formation increased NF-κB transcription leads to greatly elevated vascular cell adhesion molecule-1 expression early in lesion formation in animals on an atherogenic diet^37,38^, while eNOS protein and mRNA expression are reduced in the same regions^39^. There is strong evidence from non-atherosclerotic coronary vessels that a reduction in L-arginine availability contributes to the acute impairment of endothelium-derived, NO-mediated dilation by LDLs and especially oxLDL without altering eNOS expression. LDLs have been shown to promote uncoupling of eNOS from NO production, and increase superoxide radical generation, which further reduce NO bioavailability through peroxynitrite formation^10,11^. oxLDL also exacerbate L-arginine deficiency by increasing vascular arginase activity and thereby consuming the L-arginine that is available in the vessel wall^40^. Further, the thrombogenic inflammatory biomarker, C-reactive protein, might also contribute to impaired NO-mediated dilation *in vivo* as it has been shown to inhibit eNOS activity and upregulate NADPH oxidase and superoxide generation *in vivo*, and impair endothelium-derived, NO-mediated dilation in isolated arterioles^41^. It is interesting to note that the same authors have also shown that the endothelium-mediated NO-signaling pathway that is impaired by oxLDL infusion in normal arterioles involves iberiotoxin-sensitive large Ca^2+^-activated K channels^11^, which are important for local radial transmission of dilation. Thus, lesion formation in macro coronary vessels in SR-B1^−/−^/ApoER61^h/h^ mice appears to be preceded by impaired NO signaling of dilation, which likely involves a reduction in NO bioavailability due to uncoupling of eNOS from NO production and impaired vascular smooth muscle function by mechanisms that remain to be determined. However, impaired transmission of NO-mediated dilation to the vascular smooth muscle layer could also be due in part to changes in large Ca^2+^-activated K channel function^11^. Moreover, impairment of vascular function was found to extend from large arteries through to the arterioles involved in the maintenance of basal perfusion.

A significant novel finding of this study is that the transmission of dilatory signals along the length of the vessels and their side branches in SR-B1 deficient mice, which we assume might be through a process of conducted dilation through endothelial cell-to-cell communication, which permitted the entry of contrast agent into newly visualized vessels, was preserved. SR-B1^−/−^/ApoER61^h/h^ mice showed similar mean increases in the number of large and small vessels during ACh infusion (pre-blockade) as SR-B1^+/−^/ApoER61^h/h^ mice, although highly variable (Fig. 7). Furthermore, the NO donor SNP also increased global perfusion by the opening of newly visualised vessels in these SR-B1 deficient mice. Taken together these findings imply that while NO bioavailability appears to be reduced in coronary vessels in SR-B1^−/−^/ApoER61^h/h^ mice, NO donation was able to increase perfusion of other side branches distal to the segments observed at baseline. We speculate that these mice might maintain the ability to evoke global increases in perfusion by NO donors and other agonists in the non-occluded vessels that are only open as metabolic demand requires through EDHF-mediated pathways involving close endothelial cell-to-cell contacts, such as gap junctions^30^. Conducted dilation is considered to be predominately mediated by EDHF through longitudinal transmission of a dilatory signal to reduce the vascular resistance of side and distal resistance vessels along a vessel segment^30–32^. Notably, recently it has been shown that NO plays a facilitatory role in enhancing EDHF-conducted dilation^30^. Currently, it is not possible to test *in vivo* in the beating heart whether our observations are truly due to conducted dilation through endothelial cell-to-cell communication.

In support of earlier studies^17,20,22,28^, this study clearly shows that EDHF-mediated local increases in coronary vessel calibre (radial dilation) and increases in global perfusion through the opening of newly visualized vessels were not significantly diminished by brief exposure to the atherogenic diet in the absence of SR-B1. Interestingly, vasodilation through bradykinin, prostanoids and several EDHF pathways were not affected by acute infusion of oxLDL in normal porcine arterioles^11^. Our observations that vessel calibre, both in non-occluded segments (Fig. 5) and at the leading edge of occlusions (Fig. 8), were significantly reduced following NOS/COX blockade suggests that dilatory prostanoids most likely contribute to the maintenance of coronary perfusion in the absence of SR-B1. Since others have shown that transmission of conducted dilation along vessels involves gap junctions^30^ and small and medium conductance Ca^2+^-activated K channels^29^ it seems reasonable to suggest that these channels are likely to be important for maintaining the ability to increase perfusion in SR-B1^−/−^/ApoER61^h/h^ mice after exposure to an atherogenic diet. Nevertheless, residual dilation mediated by EDHF was absent in largely occluded segments and their adjacent upstream segments of large to small arterial vessels. This implies that loss of dilator function at the leading edges of intravascular plaques occurs as a precedent to the enlargement of the lesions. One possibility is that foam cell accumulation contributes to the localized disruption of endothelium-smooth muscle cell continuity and EDHF-mediated dilation. Nevertheless, one or more of the putative EDHF and prostanoids appear to be largely responsible for the maintenance of perfusion in this model of ischemic cardiomyopathy, and the preservation of the ability to increase global perfusion according to metabolic requirements.

As expected, this study confirmed that SR-B1^−/−^/ApoER61^h/h^ mice challenged with the atherogenic diet developed diffuse coronary lesions, spontaneous myocardial infarctions due to complete occlusion of a main coronary artery by foam cell rich plaques or thrombosis (Suppl. Fig. S6) and cardiac remodeling typical of heart failure. The partially occlusive atherosclerotic plaques found in macrovessels >100 µm ID were clearly visible as a stenosis in the *in vivo* cine-angiograms obtained from SR-B1^−/−^/ApoER61^h/h^ mice (Figs 4, 6 and Suppl. Fig. S3). Notably, we show that NO-mediated vascular dysfunction was widespread, including the resistance microvessels <100 µm ID, two weeks after exposure to hypercholesterolemic diet in the SR-B1^−/−^/ApoER61^h/h^ mice. Sustained nitrosative and oxidative stress are almost certainly a major contributor to the vascular dysfunction observed in the non-occluded arteries and arterioles of SR-B1 deficient mice in this study, and presumably diminished NO production, as myocardial nitrotyrosine expression was greatly increased according to immunohistochemical staining (Fig. 1). Furthermore, hypoxia-induced factor 1α (HIF-1α) gene expression showed a strong trend for progressive elevation after the dietary challenge in SR-B1^−/−^/ApoER61^h/h^ mice^9^. It seems unlikely that hypercholesterolemia per se is important as a contributor to the progression of vascular dysfunction in this model as cholesterol levels return to pre-treatment levels within 1 week after dietary intervention, except that HDL remains suppressed^9^. However, a limitation of this study is that we were unable to establish if oxLDL were normalized at the time of imaging 2 weeks after exposure to the Paigen diet. Therefore, we can only establish that a reduced antioxidant capacity associated with low HDL levels and SR-B1 deficiency and activation of pro-inflammatory cascades^9^ contribute to microvascular dysfunction, functional and structural rarefaction and the rapid development of ischemic cardiomyopathy and heart failure in SR-B1^−/−^/ApoER61^h/h^ mice. Diminished NO-mediated endothelial function is likely to be a key factor in many of these changes, which needs to be investigated in future studies.

In summary, microangiography utilizing synchrotron radiation enabled us to examine the involvement of impaired dilator function of the coronary microvessels in the progression of atherosclerosis and ischemic cardiomyopathy. Using this within-segment analysis technique we have shown that even short-term exposure to an atherogenic diet in SR-B1^−/−^/ApoER61^h/h^ mice led to eNOS uncoupling and widespread impairment of NO-mediated dilation, but did not affect EDHF-mediated dilation except at the upstream edges and within lesions. These findings provide a strong basis for further investigation into the pathophysiological changes that occur in the development of CAD and ischemic heart disease as synchrotron radiation also enables within-animal assessment of individual arteries during vascular stimulation *in vivo* ^25–27^.

## Materials and Methods

### Animals and Experiments at the Synchrotron

Male SR-B1^−/−^/ApoER61^h/h^ (SR-B1^−/−^) and SR-B1^+/−^/ApoER61^h/h^ (SR-B1^+/−^) mice (mixed C57Bl/6 x s129 background^8^) were bred at Osaka University with approval by the Animal Experiment Committee for this study by mating female SR-B1^+/−^/ApoER61^h/h^ mice with male SR-B1^−/−^/ApoER61^h/h^ mice, since female SR-B1^−/−^/ApoER61^h/h^ mice have been shown to be infertile. All imaging experiments were conducted at Beamline 28B2 of SPring-8, Japan Synchrotron Radiation Research Institute, Hyogo, Japan with approval of the SPring-8 Animal Experiment Review Committee and the National Cerebral and Cardiovascular Center Animal Experiment Committee. All methods were performed in accordance with the relevant guidelines and regulations of the Physiological Society of Japan.

Under sodium pentobarbital anesthesia (1:10 diluted solution, 50 mg/kg i.p.) mice were intubated, artificially ventilated (40% oxygen, Harvard Hugo-Sachs Minivent, 180 cycles/min at 7 µl/g body mass) and the right carotid artery cannulated with a Instech-Solomon FunnelCath™ polyurethane catheters (PUFC-C30–10) connected to a 22 G needle hub for contrast injection. Prior to experiments both 1.2 F and 3 F sections of the catheter are shortened, reducing the 1.2 F segment to ~42 mm length (Fig. S1). To insert the polyurethane catheter to the aortic valve in the SR-B1^−/−^/ApoER61^h/h^ mouse strain required the use of an improvised 24 G guidewire from a pediatric radiography cannula, placing the tip at the entrance of the aortic valve. Body temperature was maintained at 37 °C using a rectal thermistor coupled with a thermostatically controlled heating pad. Anesthesia level was maintained via additional intraperitoneal boluses of pentobarbital (20 mg/kg/h). Sodium lactate was administered intravenously via the right jugular vein to maintain body fluids (0.2 ml/hr). Arterial pressure was intermittently recorded via a disposable pressure transducer (MLT0699, AD Instruments, NSW, Australia) from carotid arterial line. The analogue arterial pressure signal was digitized at 1000 Hz and recorded using CHART software (v5.4.1, AD Instruments, NSW, Australia) to determine mean arterial pressure (MAP) and heart rate, simultaneous with cine-recordings of the camera during each treatment period.

### Cine-Angiography Protocol

Each mouse was then moved into the X-ray hutch for contrast angiograms, with the long axis of the heart aligned perpendicular with the horizontal X-ray beam and SATICON detector system (Hitachi Denshi Techno-system, Ltd., Tokyo, Japan and Hamamatsu Photonics, Shizuoka, Japan), described previously^25^. Iodinated contrast medium (Iomeron 350; Bracco-Eisai Co. Ltd, Tokyo, Japan with 160 U/ml heparin) was remotely injected intrarterially as a bolus (50–150 µl at 0.4 ml/s over 1 s using a Harvard PHD200 pump, Harvard Apparatus, Holliston MA, using a Terumo 3 ml syringe) into the aorta and cine-angiograms^25^ were obtained over 3 s whilst maintaining continuous ventilation. Arterial pressure and heart rate was recorded until immediately before the angiogram acquisition when the three-way valve was closed to the pressure transducer for contrast administration. After image acquisition the valve was closed to the syringe pump to allow continued blood pressure recording. At least 10 minutes was allowed for renal clearance of contrast between imaging scans. During each cine-scan, monochromatic X-rays at 33.2 keV (energy bandwidth 20–30 eV) and a flux ~10^10^ photons/mm^2^/s passed through the chest of the mouse and was recorded on the X-ray detector (Hitachi Denshi Techno-System, Ltd., Tokyo, Japan) at 30 frames/s at 10-bit resolution for ~3 s intervals. For each cine-scan 100 frames were recorded with a shutter open time of 0.8–1.0 ms/frame. The detector features a 9.5 μm equivalent pixel size that captures a 9.5 × 9.5 μm input field with images stored in 1024 × 1024 pixel format. Periodic inspection between angiograms confirmed that the coronary vasculature cleared of contrast agent during this time.

### Experimental Protocol

Endothelium-dependent and –independent vasodilatory responses were recorded in SR-B1^−/−^/ApoER61^h/h^ (n = 8) and SR-B1^+/−^/ApoER61^h/h^ (n = 8) mice. This was achieved by recording coronary angiograms during infusions of vehicle (sodium lactate 0.2 ml/hr), acetylcholine (ACh, 5.0 μg/kg/min, 0.2 ml/h) and sodium nitroprusside (SNP 5.0 μg/kg/min). Further, imaging was repeated 30 minutes after combined inhibition of NOS with Nω-nitro-l-arginine methyl ester (L-NAME, 50 mg/kg iv. bolus in 100 μl) and cyclooxygenase (COX) with sodium meclofenamate (3 mg/kg iv. bolus in 100 μl), and finally post blockade during a second infusion of ACh. At the termination of experiments mice were killed and their hearts removed.

### Histology, Immunohistochemistry and Western Blotting

Hearts were fixed in 4% paraformaldehyde and stored in 70% ethanol. All histological and immunohistochemical sections were imaged using the Aperio ScanScope XT Slide Scanner system (Aperio Technologies, Inc., CA, USA). Nitrotyrosine and eNOS protein staining was performed to investigate nitrosative stress and total eNOS expression across the myocardium, including the coronary vasculature, myocardium and extracellular matrix. First sections were subjected to heat-mediated antigen retrieval, followed by incubation with 3% H_2_O_2_ for 15 min at room temperature and washing three times with PBS (pH 7.4) for 5 min each. Nonspecific protein binding was blocked with serum free protein block (Dako, Golstrup, Denmark, 10 min for nitrotyrosine and 30 min for eNOS). The sections were then incubated with the primary antibody overnight at 4 °C (anti-nitrotyrosine 1:500, Millipore 6-284 and anti-eNOS 1:500, Becton-Dickson 610297). Following this, sections were incubated with goat anti-rabbit horseradish peroxidase (HRP) (Dako, Golstrup, Denmark) for 60 min at room temperature and developed using diaminobenzidine (Vector Laboratories, Inc, Burlingame, CA, USA) and finally counter-stained with haematoxylin. Perivascular fibrosis expression was determined as the ratio of the area of fibrosis immediately surrounding the intramyocardial blood vessel walls to the total area of the vessel for coronary arteries and arterioles stained with Masson’s trichrome. The proportional area of the positively stained protein for nitrotyrosine and total eNOS were automatically quantified using the Positive Pixel Count v9 algorithm on Aperio Imagescope (v11.0.2.725, Aperio Technologies) in ten non-overlapping fields that were randomly selected across the myocardium in two non-adjacent slices from the middle of the heart per mouse. Only strongly positive pixels were included in nitrotyrosine stained section analyses.

Hearts from separate mice from both groups (n = 4/group) were frozen for eNOS Western blotting. Myocardial tissue was homogenised with lysis buffer containing protease inhibitors and protein concentrations determined by BCA assay method. Proteins (30 μg) were separated on 10% SDS gels (Bio-Rad, CA, USA) and subsequently transferred to nitrocellulose membranes. Non-specific binding was blocked with Blocking One solution (Nacalai Tesque 03953-95, Kyoto, Japan) for 1 hour at room temperature. Membranes were then incubated with primary antibody (1:1000) for total eNOS (Thermo-Fisher PA1-037), p-eNOS Ser1177 (PA5-17917), p-eNOS Thr495 (PA5-17706) and β-actin (Santa Cruz, Sc-47778) overnight at 4 degrees. After washing three times, membranes were incubated with secondary antibody (anti-mouse Cell Signaling Technology 7045, 1:5000 for 1 h, room temperature) and then the bound antibody visualised with SuperSignal^TM^ West Dura Extended Duration Substrate (Thermo-Fisher 34075) using a chemiluminescence imaging system (ImageQuant LAS 4000mini, Tokyo, Japan). Optical density of total eNOS and p-eNOS were normalised to β-actin band intensity.

### Assessment of Vessel Internal Diameter

Quantitative analysis of vessel internal diameter (ID) was based on measurements from the middle of discrete vessel segments in individual cine-angiograms using Image-J (v1.41, NIH, Bethesda, USA). Angiograms underwent median filtering (2-pixel radius) before ID determination. Arterial vessels were categorized according to their branching order (1^st^, 2^nd^, 3^rd^ and 4^th^). Reported results for vessel ID and vessel number during drug infusions are expressed as percentage change from baseline to account for differences in absolute baseline vessel ID and vessel number between groups. Vessel recruitment was determined as the percentage change in visualized vessel number from baseline during each treatment for the same field of view.

Recently we have shown that analysis of local vessel calibre changes and changes in the number of visualized vessels during agonist stimulation enables the assessment of local endothelial function and the capacity to increase flow by distal dilation^42^. Here we assume that vessel calibre changes reflect the state of local endothelial function and the local balance of dilator and constrictor factors, whereas a change in the number of perfused vessels that are angiographically visible during stimulation reflects the ability to increase global myocardial perfusion with changing metabolic requirements through longitudinal transmission of dilation along the length of vessels.

### Statistical Analysis

Data are expressed as mean ± SEM unless otherwise stated. For each individual animal, the mean vessel ID or mean change in vessel ID (%) of each branching order was used for group comparisons^25^. One-way and two-way ANOVA with Holms-Sidak post hoc tests were carried out to assess within and between group differences, followed by a two-tailed Student t-test to determine statistical significance between groups and one-tailed t-test for differences from baseline using Prism 7 software (GraphPad). For all analyses values of p < 0.05 were deemed significant.

### Data Availability

All data generated or analysed during this study are included in this article or its Supplementary Information file.

## Electronic supplementary material

Supplementary Information
