# Supplementary material for: Widespread Coronary Dysfunction in the Absence of HDL Receptor SR-B1 in an Ischemic Cardiomyopathy Mouse Model
Source: Sci Rep. 2017 Dec 22;7:18108. doi: 10.1038/s41598-017-18485-6 (PMC5741771; doi:10.1038/s41598-017-18485-6)
Supplement: Supplementary file 1 — Supplementary Information [file 41598_2017_18485_MOESM1_ESM.pdf]

Widespread Coronary Dysfunction in the Absence of HDL Receptor SR-B1 in an Ischemic Cardiomyopathy Mouse Model

James T Pearson[1,2,3,4*], Misa Yoshimoto[4#], Yi Ching Chen[2¶], Rohullah Sultani[2¶], Amanda J Edgley[2,5], Hajime Nakaoka[6], Makoto Nishida[6], Keiji Umetani[7], Mark T Waddingham[5], Hui-Ling Jin[4], Yuan Zhang[5], Darren J Kelly[5], Daryl O Schwenke[8], Tadakatsu Inagaki[4], Hirotsugu Tsuchimochi[4], Issei Komuro[9], Shizuya Yamashita[10,11], Mikiyasu Shirai[4]

[1]*Monash Biomedical Imaging Facility, Melbourne, Victoria, Australia*
[2]*Department of Physiology, Monash University, Melbourne, Victoria, Australia*
[3]*Australian Synchrotron, Melbourne, Victoria, Australia*
[4]*National Cerebral and Cardiovascular Center Research Institute, Suita, Osaka, Japan*
[5]*St Vincent's Hospital, University of Melbourne, Melbourne, Victoria, Australia*
[6]*Department of Cardiovascular Medicine, Graduate School of Medicine, Osaka University, Suita, Osaka, Japan*
[7]*Japan Synchrotron Radiation Research Institute, Harima, Hyogo, Japan*
[8]*Department of Physiology – HeartOtago, University of Otago, Dunedin, New Zealand*
[9]*Department of Cardiovascular Medicine, Graduate School of Medicine, University of Tokyo, Tokyo, Japan*
[10] *Departments of Community Medicine and Cardiovascular Medicine, Graduate School of Medicine, Osaka University, Suita, Osaka, Japan*
[11]*Rinku General Medical Center, Izumisano, Osaka, Japan*

[#]Current Address: Department of Health Sciences, Nara Women's University, Nara, Japan

*Corresponding author:

Prof James T Pearson
Department of Cardiac Physiology,
National Cerebral and Cardiovascular Center Research Institute,
5-7-1 Fujishirodai Suita-shi 565-8565, Japan
Tel: +81668335012
Email: jpearson@ncvc.go.jp

Supplementary information

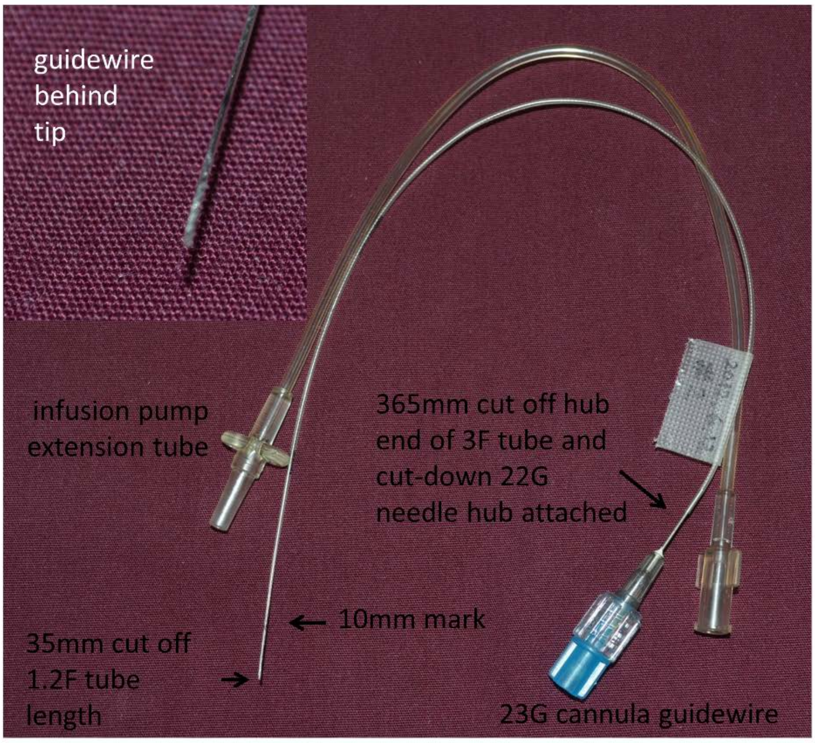

Figure 1. Polyurethane cannula modified for angiography in anesthetized mice. For optimal contrast delivery the FunnelCath™ is shortened as illustrated to reduce resistance and improve bolus delivery of the higher viscosity iodine contrast agent. A pediatric guidewire is cut to match catheter length and used for catheter insertion procedure after priming the catheter with heparinised saline (12 Units/ml). Once the catheter is inserted to the 10mm mark, approximately at the aortic valve in C57Bl6 mice (12-13 mm in larger BALBc mice), the guidewire is pulled out and cannula hub connected to a three-way valve. The blood pressure transducer is then connected to the side arm of the valve for recording arterial pressure in between angiogram acquisitions (arm is closed during iodine injection). Finally, once the mouse is placed securely in the synchrotron hutch a short infusion extension tube pre-primed with iodinated contrast agent is connected between the arterial cannula (remaining arm of the three-way valve) and syringe pump to commence imaging with remote bolus injection of iodine contrast agent.

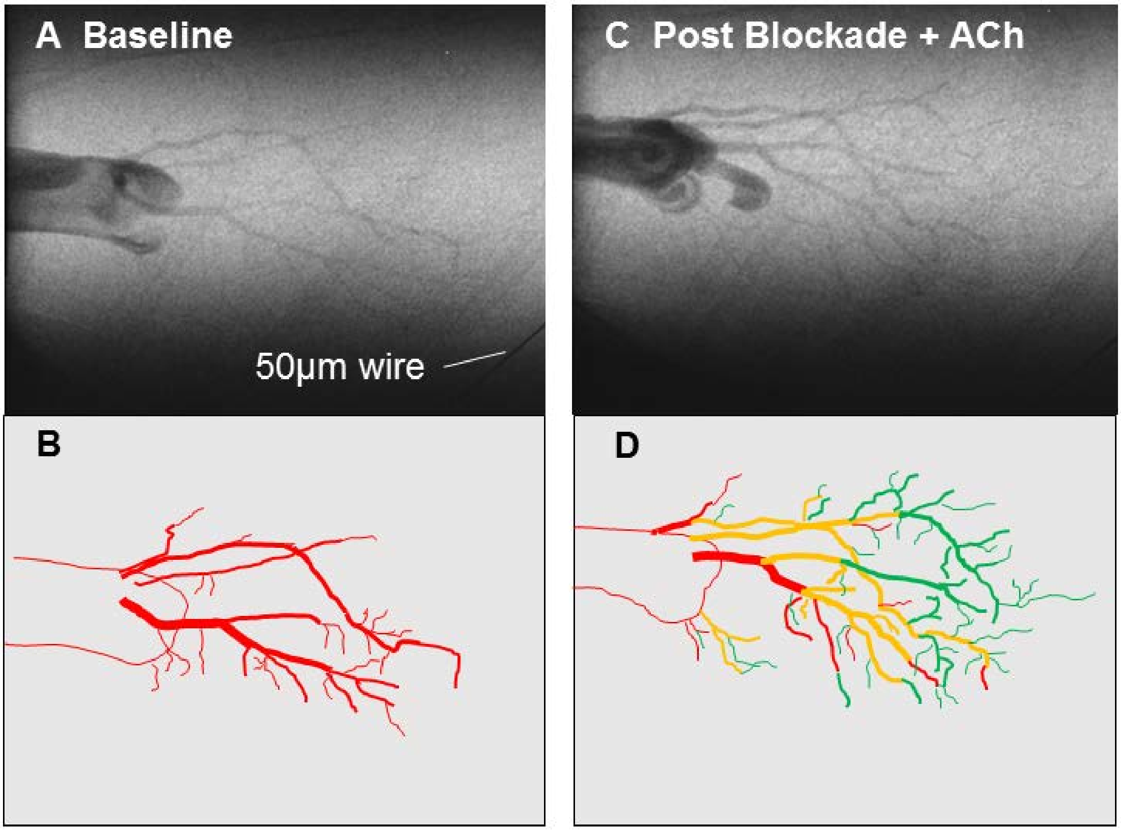

Figure 2. Typical angiograms collected from pentobarbital (50 mg/kg) anesthetised male C57BL6/J mouse clearly shows the differences between local radial dilation as opposed to increases in newly visualised vessels. Coronary angiogram at baseline (**A**) and during ACh stimulation (5 µg/kg/min) following NOS/COX inhibition (L-NAME 50mg/kg and Meclo 3mg/kg) (**C**). Red lines indicate the extent of visible vessels at baseline (**B**). During ACh stimulation post blockade vessels that were visible at baseline and increased in vessel calibre are shown in orange (local dilation), while vessels that did not change in vessel calibre are red (**D**). Newly visualised vessels (resulting in an increase in visible vessel number) are shown in green.

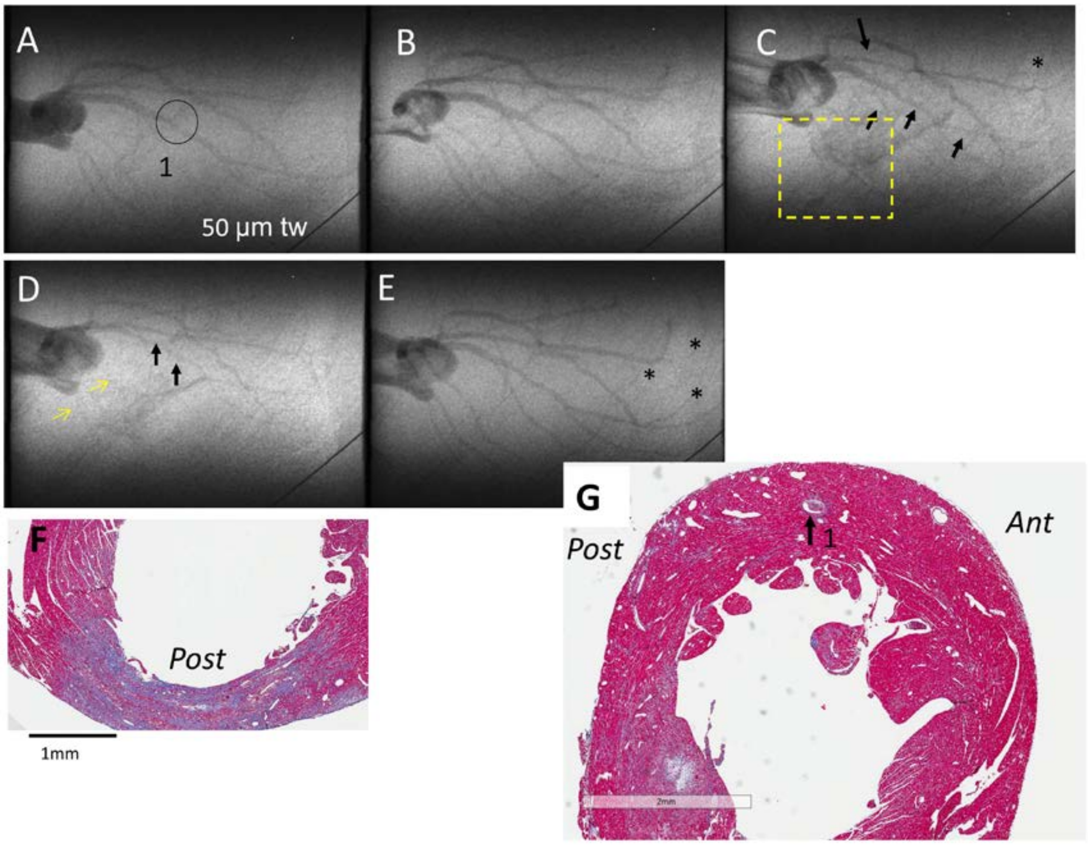

Figure 3. Vascular responses in an SR-B1$^{-/-}$/ApoER61$^{h/h}$ mouse. Largely normal anterior wall circulation with one clear stenosis (1) at baseline (**A**). Large vessel caliber maintained while medium-small vessels dilated during ACh infusion (**B**). One main artery and several branches showing constriction in response to NO donor (black arrows) (**C**), and further constriction in vessels to the posterior wall (yellow dashed box). Widespread constriction following NOS/COX blockade with L-NAME and meclofenamate (yellow arrows indicate posterior wall vessels)(**D**). Flow largely restored to baseline during ACh stimulation following blockade (**E**). Basal section (**F**) and mid LV section (**G**) stained with Masson's trichrome showing a posterior wall infarct and a largely occluded coronary artery, respectively. Asterisks indicate newly visualized vessel segments.

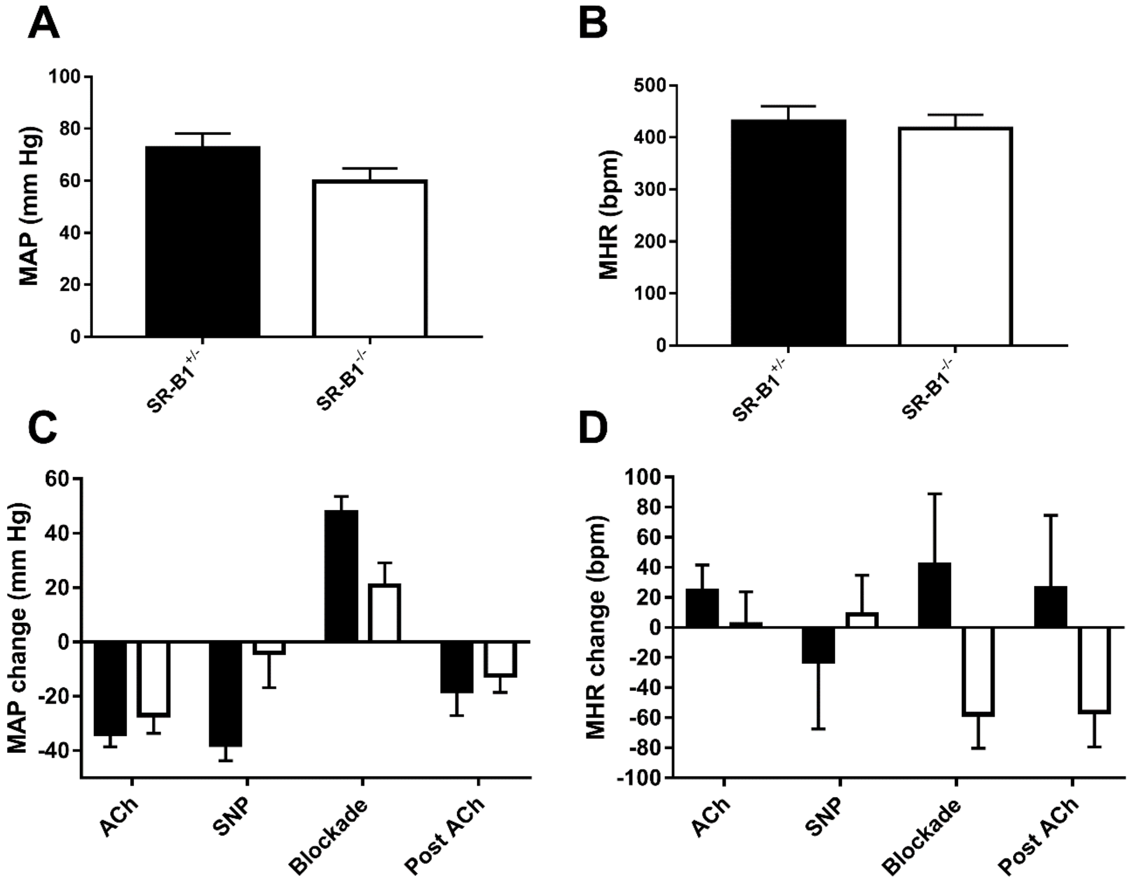

Figure 4. Mean arterial pressure (MAP) and mean heart rate (MHR) and their change during treatment periods. Changes in MAP and MHR during infusion of ACh, SNP, L-NAME + meclofenamate and Post (blockade) + ACh relative to vehicle baseline. SR-B1$^{-/-}$/ApoER61$^{h/h}$, n=4-8 and SR-B1$^{+/-}$/ApoER61$^{h/h}$, n=8. Values expressed as mean±SEM. **p<0.01, ***p<0.001 vs. SR-B1$^{+/-}$/ApoER61$^{h/h}$.

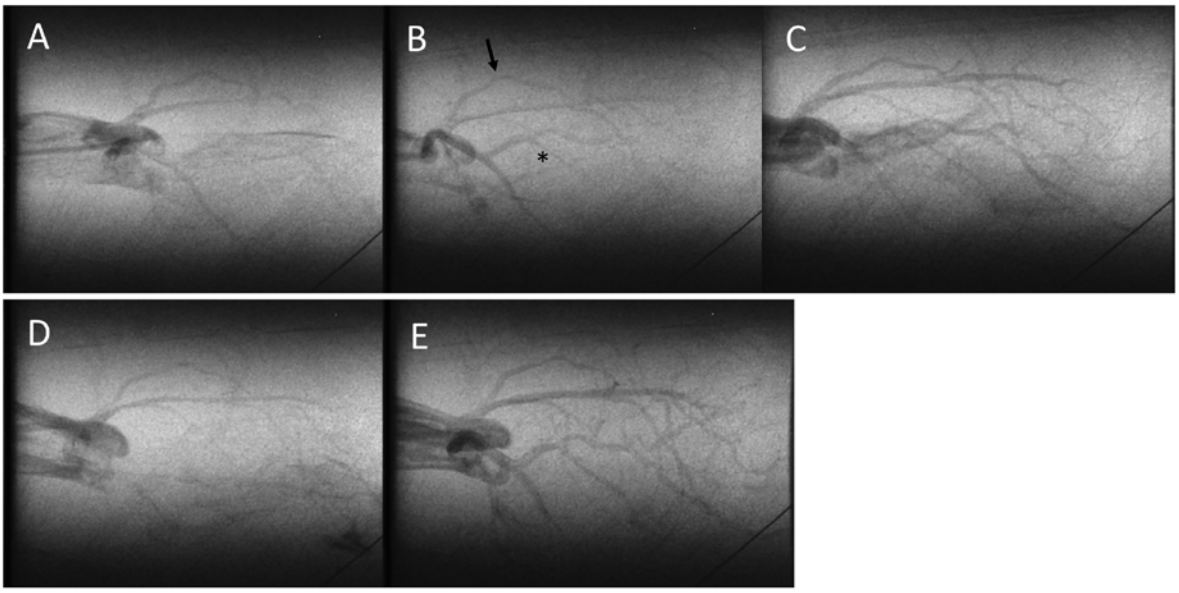

Figure 5. Vascular responses of a SR-B1$^{-/-}$/ApoER61$^{h/h}$ mouse. Baseline response to lactate infusion (**A**). Large vessels either maintained calibre similar to baseline, or were constricted during ACh infusion (arrow), but showed distal dilation and new branches (*) (**B**). Dilation of distal microvessels during SNP infusion with little change in large artery calibre (**C**). Widespread constriction of microvessels following administration of L-NAME and meclofenamate to block NOS and COX (**D**). Dilation of large and small arteries with some recruitment of newly visualised microvessels during ACh infusion post NOS/COX blockade (**E**).

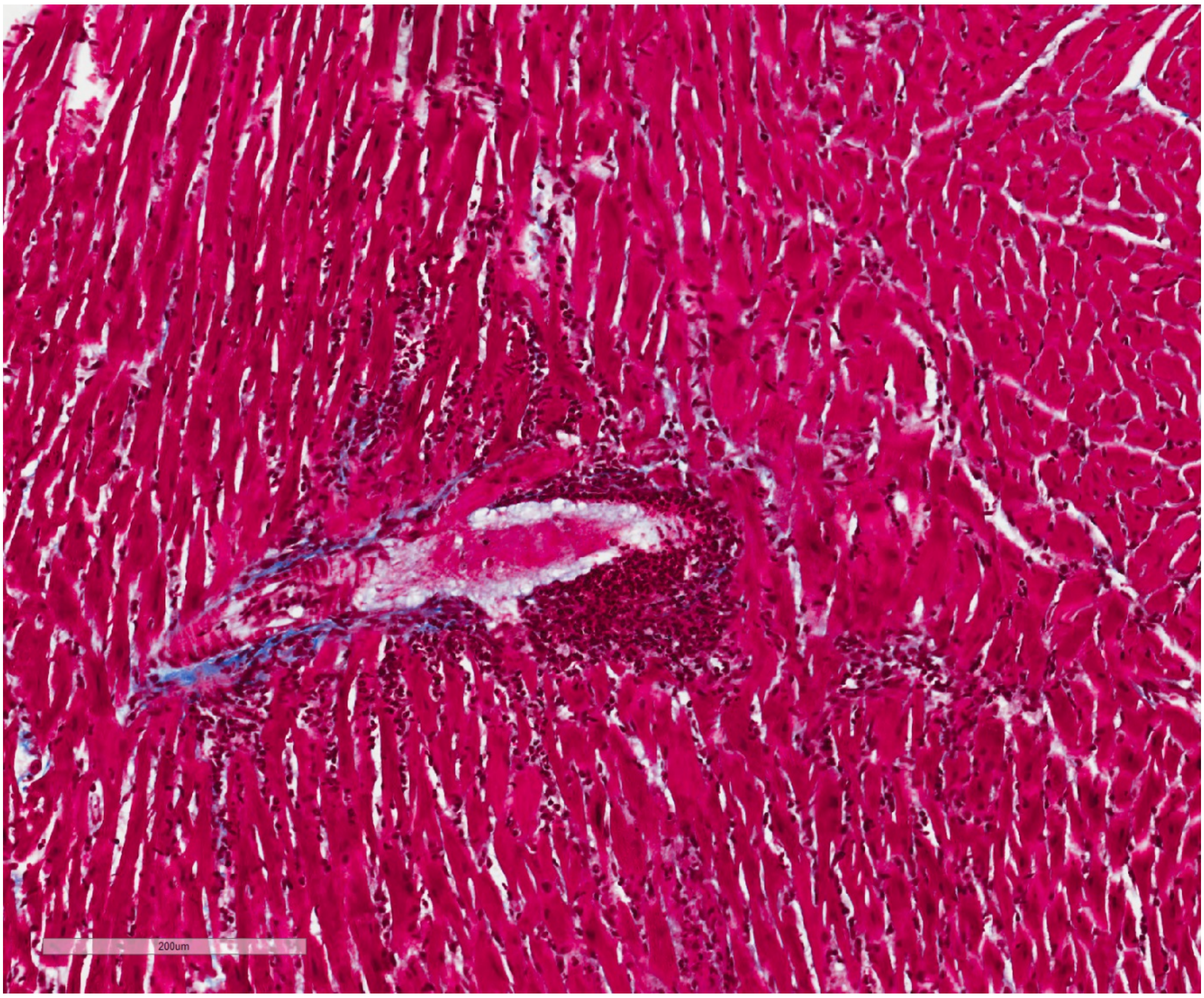

Figure 6. Recent thrombus formation at a branching point of a small penetrating artery in a SR-B1$^{-/-}$/ApoER61$^{h/h}$ mouse. An example of a small artery in the subepicardial region showing a recent thrombosis at a site of foam cell accumulation. Immune cell accumulation is evident in the microvessels and interstitial spaces surrounding the thrombosis. Masson's trichrome stained section (20X objective).
